# Supplementary material for: Intra-Patient Evolution of HIV-2 Molecular Properties
Source: Viruses. 2022 Nov 4;14(11):2447. doi: 10.3390/v14112447 (PMC9698092; doi:10.3390/v14112447)
Supplement: Supplementary file 1 [file viruses-14-02447-s001.zip › viruses-1964323-supplementary.pdf]

Intra-Patient Evolution of HIV-2 Molecular Properties

Angelica A. Palm[1, 2], Joakim Esbjörnsson[2, 3], Anders Kvist[4], Fredrik Månsson[2], Antonio Biague[5], Hans Norrgren[4], Marianne Jansson[1] and Patrik Medstrand[2]

[1] Department of Laboratory Medicine, Lund University, Lund, Sweden
[2] Department of Translational Medicine, Lund University, Lund, Sweden
[3] Nuffield Department of Medicine, University of Oxford, Oxford, UK
[4] Department of Clinical Sciences, Lund University, Lund, Sweden
[5] National Public Health Laboratory, Bissau, Guinea-Bissau

SUPPLEMENTARY FILE 1

1. Material and methods

1.1. Study population

This study included individuals from a large cohort of police officers in Guinea-Bissau, West Africa which was formed in 1990 and has been described in detail elsewhere [1, 2]. At inclusion, and at follow-up visits scheduled with an interval of 12-18 months, individuals were examined and a blood sample was collected. The civil war in 1998-1999 temporarily closed inclusion from June 1998 until the end of 2000 but annual visits of previously included patients was resumed in July 2000. The cohort was followed routinely until February 2011 when the cohort was closed. However, in September 2013 selected individuals from the cohort were asked to participate in a special sampling round, including a clinical examination and the collection of a blood sample. HIV testing was performed at the National Public Health Laboratory (LNSP), Bissau, as previously described [2]. The cohort includes 438 HIV-2 infected individuals of whom 83 individuals have an estimated date of seroconversion, defined as the midpoint between the last HIV-2 seronegative and the first seropositive sample. Individuals with long follow-up series, including both CD4% measurements and available plasma samples were considered for inclusion into the study. However, our initial goal was to conduct this study by including only individuals with estimated dates of infection. Thus, amplification of viral RNA was attempted on plasma samples from seroincident individuals for whom three or more longitudinal plasma samples were available (n=19). As expected, due to the general low viral load in HIV-2 infections, amplification was only successful for a minority of samples and only seven individuals with two or more successfully amplified longitudinal samples could be included in the study. We therefore decided to also include seroprevalent individuals in the study. Due to the difficulties amplifying viral RNA only individuals with more than six available plasma samples were considered for inclusion. Amplification was attempted on samples from 19 individuals and successful amplification of two or more longitudinal samples was achieved for nine individuals. Taken together, amplification was attempted on samples from 38 individuals, but only 16 individuals fulfilled the inclusion criteria of two amplified longitudinal samples (total 53 samples) (Table S1).

The 16 included individuals have previously been classified as faster or slower progressors based on three different stratifications [3]. The three stratifications were all based on longitudinal CD4% measurements, and faster and slower progressors were defined by a value above or below the mean of all included individuals. The first stratification, referred to as CD4% decline rate, was based on CD4% decline over time during the asymptomatic, treatment-naïve phase of infection and classified nine individuals as faster progressors and seven individuals as slower progressors. As not only CD4% decline rate but also the level of CD4% may influence disease outcome [4], we also included this parameter to define different progressor groups. In the second stratification, referred to as CD4% level, we calculated the CD4% level at the midpoint in time between the first and last amplified sample using the regression coefficient generated in the first stratification. This stratification classified 10 individuals as faster progressors and six individuals as slower progressors. The third stratification, referred to as the combined coefficient, combined the two previous stratifications, taking both CD4% decline rate and level into consideration. The regression coefficient and CD4% values were transformed and rescaled to have equal influence on the combined coefficient. The combined coefficient generated identical groups as the CD4% level, but the individual rankings varied within the groups.

1.2. Amplification and sequencing

Viral RNA was extracted from 200 µl of plasma by disruption in 2000 µl Qiazol, using the miRNeasy micro kit (Qiagen, Stockholm, Sweden) with minor modifications to the manufacturer's instructions. RNA was loaded onto an RNeasy MinElute Spin column in the presence of 15 µg carrier RNA and an on-column DNase-treatment (Qiagen) was used to

remove DNA. The RNA was washed and subsequently eluted in 22 µl RNase-free H_2O. Amplification was performed on 9.5 µl RNA using the SuperScript III One-Step RT-PCR System with Platinum Taq DNA polymerase followed by a semi-nested PCR using Platinum Taq High Fidelity (both from Invitrogen, Copenhagen, Denmark). One-Step PCR was performed using primers KH2_OF (5´-GAGACATCAATAAAACCATGTGTC -3´) and TH2_OR (5´- TTCTGCCACCTCTGCACTAAAGG-3´) and primers KH2_OF and KH2_OR (5´-ACCCAATTGAGGAACCAAGTCA-3´) were used for semi-nested PCR [5, 6]. Following the initial cDNA synthesis for 30 min at 50ºC, the PCR conditions were identical for One-Step PCR and nested PCR: initial denaturation for 2 min at 94ºC, 40 cycles of 15 s at 94ºC, 30 s at 50ºC, 1 min at 68ºC and a final elongation step for 5 min at 68ºC. Amplification resulted in an approximate 935 nucleotide (nt) fragment including the entire V1-C3 region of *env* and small fractions of C1 and V4 regions (nucleotides 6986 to 7920 in BEN; GenBank accession number M30502). Molecular cloning of the amplified fragments was performed by BaseClear BV, (Leiden, The Netherlands), using a pCR2.1-TOPO-TA vector. Routinely, 12 individual clones were sequenced in both directions using conventional M13 primers.

1.3. Sequence analysis

Sequences were manual editing and aligned using CodonCode Aligner v1.5.2 (CodonCode Corporation, Dedham, USA) and MEGA 5 (by use of the Clustal W algorithm), respectively [7]. A pairwise homoplasy index (PHI) test using an exhaustive and iterative search algorithm was used to identify putative intra-patient recombinant sequences (the Perl script for the iterative search is available from the authors upon request). Recombinant sequences were removed in a progressive manner until the p-value was > 0.05.

2. Results

2.1. Mixed model analyses of HIV-2 evolution based on the CD4% decline rate stratification

The individuals included in this study were previously stratified into faster or slower progressors, based on CD4% dynamics [3]. Faster and slower progressors were defined based on three different stratifications; CD4% decline rate, CD4% level and a combined coefficient (Table 1, Supplementary file 1 Material and methods, and [3]). Previously, we found a strong association between evolutionary rate and the CD4% level and the combined coefficient stratifications but not with the CD4% decline rate. Therefore, the main focus of this study, and the results presented in the main text, are for the CD4% level and the combined coefficient. However, all analyses included in the main text were repeated using the CD4% decline rate stratification and results are presented below.

The relationship between each HIV-2 molecular property (diversity, PNGS, length and charge) and CD4% was initially analyzed using a mixed model including an interaction term between progressor group and CD4% (thus allowing each progressor group to have a different slope for the change of the property with declining CD4% levels). However, the interaction was not significant, i.e. there were no significant differences between groups in the change of each property with declining CD4%, for any of the analyzed properties (Table S3). Hence, we removed the interaction from the model for all subsequent analyses. Next we tested for differences between progressor groups in the estimated mean level of each property. A single significant difference was noted (Table S3): slower progressors had a higher mean number of PNGS in the V1-C3 region compared with faster progressors (net difference = 1.3 PNGS, p = 0.049). Note that since the interaction term was removed from the model, the estimated net difference in the property between groups was identical regardless of CD4% level. The number of PNGS decreased significantly with 0.08 PNGS for every percent drop of CD4 (p = 0.047 for test of slope different from zero, Table S3).

We also analyzed the evolution of each molecular property over time. Since the timing of sampling in relation to disease progression were not aligned between patients we only tested

for differences in the rate of change of each property over time, and not for differences in level, between progressor groups. The interaction between progressor group and time was not significant for any of the analyzed properties (Table S4), i.e. there were no significant differences between groups in the rate of change of any property over time. When the analyses were repeated excluding the interaction, assuming a common slope for all individuals, we found that diversity increased significantly by 7.73×10^{-4} substitutions/site/year ($p = 0.042$).

2.2. Coreceptor use

In the main text we describe the identification of eight sequences where viruses were predicted to use CXCR4 by Geno2Pheno[corecptor-hiv-2] [8] (Table 4). The individuals that harbored these sequences were from both progressor groups. Thus, no association between coreceptor use and progressor group could be identified using the CD4% decline rate stratification.

3. References

1. Norrgren H, Andersson S, Naucler A, Dias F, Johansson I, Biberfeld G: HIV-1, HIV-2, HTLV-I/II and Treponema pallidum infections: incidence, prevalence, and HIV-2-associated mortality in an occupational cohort in Guinea-Bissau. J Acquir Immune Defic Syndr Hum Retrovirol 1995, 9**:**422-428.
2. Mansson F, Biague A, da Silva ZJ, Dias F, Nilsson LA, Andersson S, Fenyo EM, Norrgren H: Prevalence and incidence of HIV-1 and HIV-2 before, during and after a civil war in an occupational cohort in Guinea-Bissau, West Africa. AIDS 2009, 23**:**1575-1582.
3. Palm AA, Lemey P, Jansson M, Mansson F, Kvist A, Szojka Z, Biague A, da Silva ZJ, Rowland-Jones SL, Norrgren H, et al: Low Postseroconversion CD4(+) T-cell Level Is Associated with Faster Disease Progression and Higher Viral Evolutionary Rate in HIV-2 Infection. mBio 2019, 8;10(1):e01245-18.
4. Esbjornsson J, Mansson F, Kvist A, Isberg PE, Nowroozalizadeh S, Biague AJ, da Silva ZJ, Jansson M, Fenyo EM, Norrgren H, Medstrand P: Inhibition of HIV-1 disease progression by contemporaneous HIV-2 infection. N Engl J Med 2012, 367**:**224-232.
5. Barroso H, Taveira N: Evidence for negative selective pressure in HIV-2 evolution in vivo. Infect Genet Evol 2005, 5**:**239-246.
6. MacNeil A, Sankale JL, Meloni ST, Sarr AD, Mboup S, Kanki P: Long-term intrapatient viral evolution during HIV-2 infection. J Infect Dis 2007, 195**:**726-733.
7. Tamura K, Peterson D, Peterson N, Stecher G, Nei M, Kumar S: MEGA5: molecular evolutionary genetics analysis using maximum likelihood, evolutionary distance, and maximum parsimony methods. Mol Biol Evol 2011, 28**:**2731-2739.
8. Doring M, Borrego P, Buch J, Martins A, Friedrich G, Camacho RJ, Eberle J, Kaiser R, Lengauer T, Taveira N, Pfeifer N: A genotypic method for determining HIV-2 coreceptor usage enables epidemiological studies and clinical decision support. Retrovirology 2016, 13**:**85.

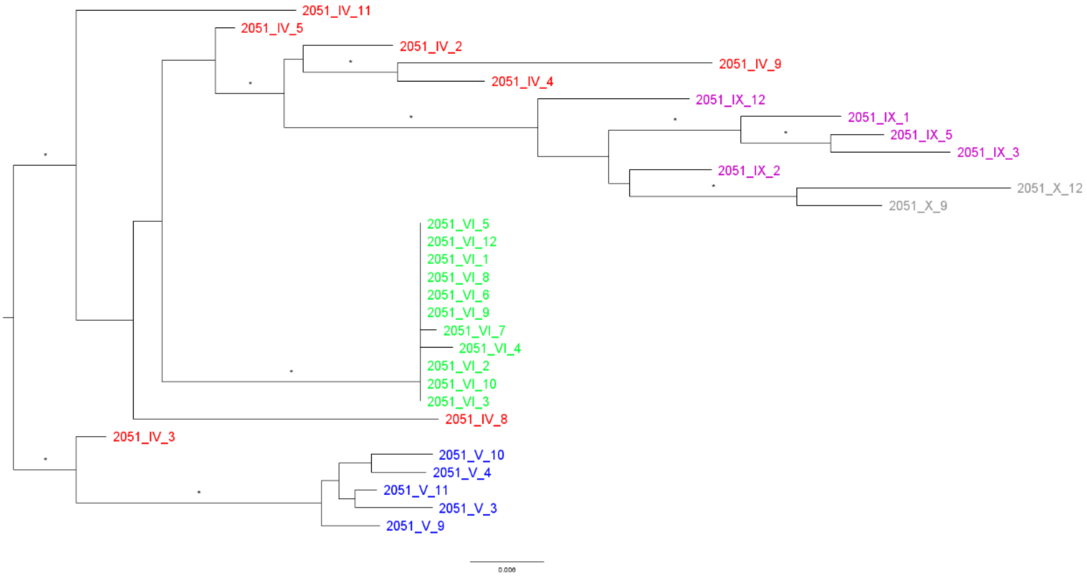

2051_IV_11
2051_IV_5
2051_IV_2
2051_IV_9
2051_IV_4
2051_IX_12
2051_IX_1
2051_IX_5
2051_IX_3
2051_IX_2
2051_X_12
2051_X_9
2051_VI_5
2051_VI_12
2051_VI_1
2051_VI_8
2051_VI_6
2051_VI_9
2051_VI_7
2051_VI_4
2051_VI_2
2051_VI_10
2051_VI_3
2051_IV_8
2051_IV_3
2051_V_10
2051_V_4
2051_V_11
2051_V_3
2051_V_9
0.006

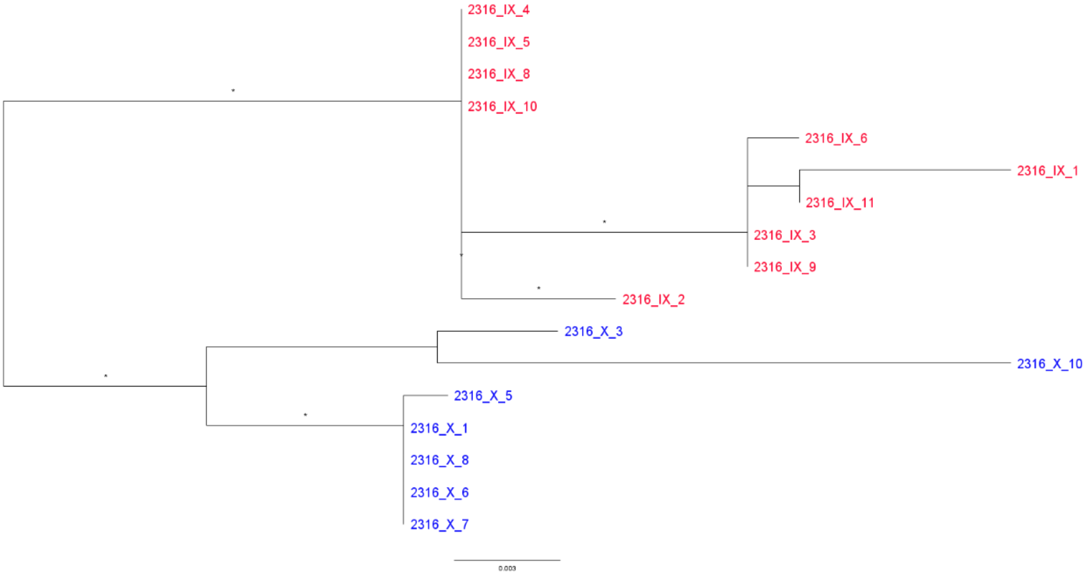

2316_IX_4
2316_IX_5
2316_IX_8
2316_IX_10
2316_IX_6
2316_IX_1
2316_IX_11
2316_IX_3
2316_IX_9
2316_IX_2
2316_X_3
2316_X_10
2316_X_5
2316_X_1
2316_X_8
2316_X_6
2316_X_7
0.003

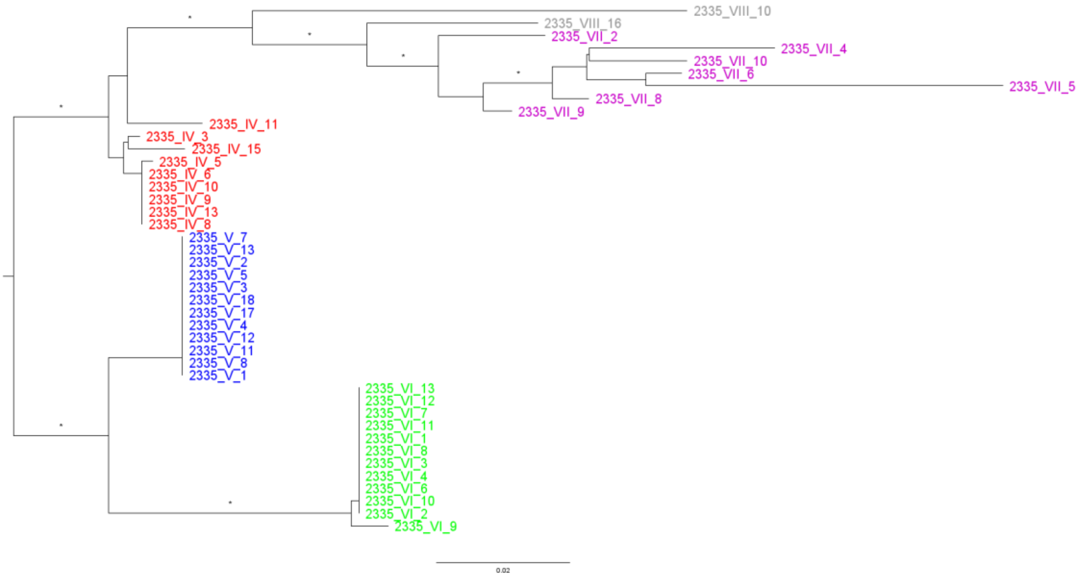

2335_VIII_10
2335_VIII_16
2335_VII_2
2335_VII_4
2335_VII_10
2335_VII_6
2335_VII_5
2335_VII_8
2335_VII_9
2335_IV_11
2335_IV_3
2335_IV_15
2335_IV_5
2335_IV_6
2335_IV_10
2335_IV_9
2335_IV_13
2335_IV_8
2335_V_7
2335_V_13
2335_V_2
2335_V_5
2335_V_3
2335_V_18
2335_V_17
2335_V_4
2335_V_12
2335_V_11
2335_V_8
2335_V_1
2335_VI_13
2335_VI_12
2335_VI_7
2335_VI_11
2335_VI_1
2335_VI_8
2335_VI_3
2335_VI_4
2335_VI_6
2335_VI_10
2335_VI_2
2335_VI_9
0.02

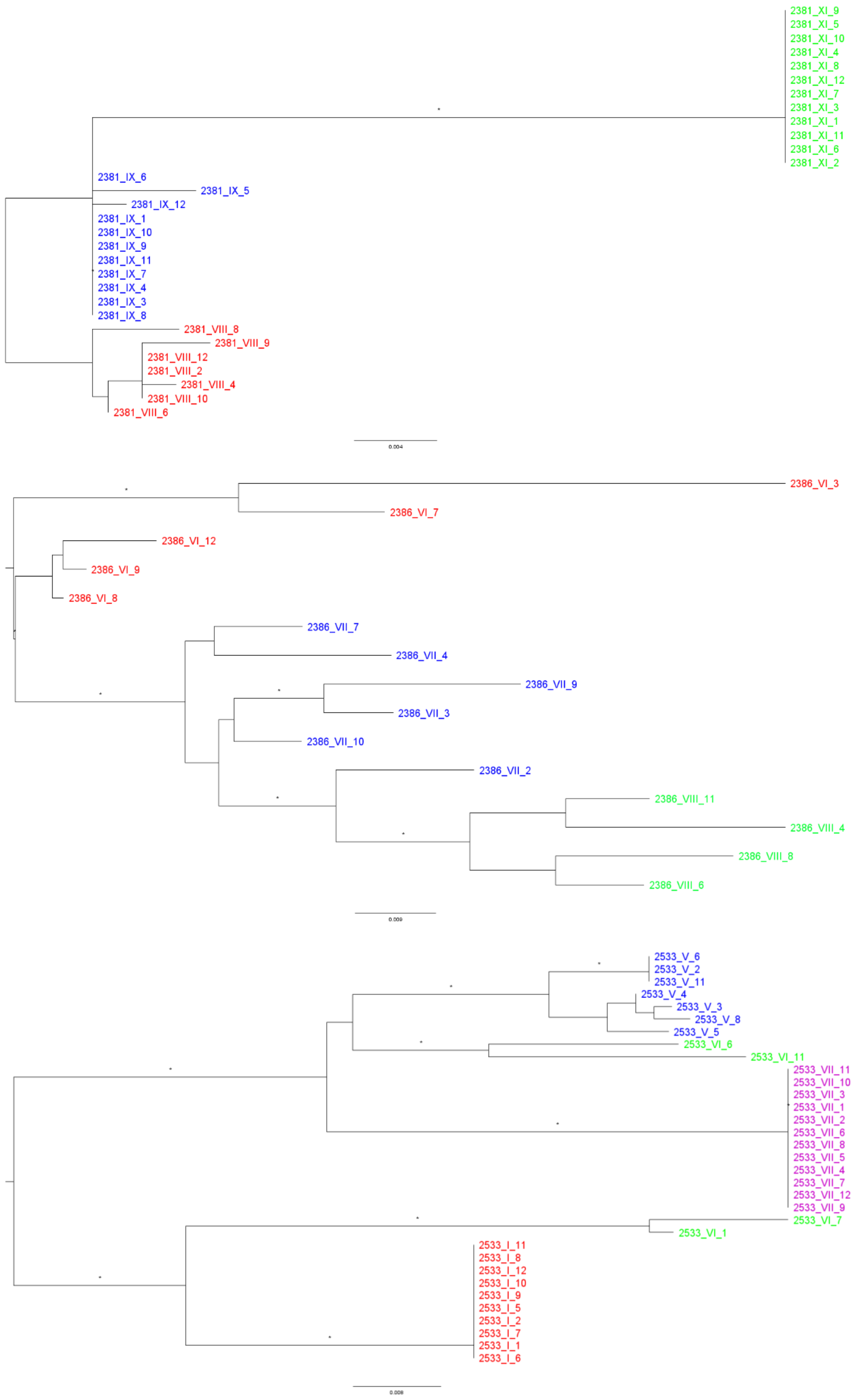

2381_XI_9
2381_XI_5
2381_XI_10
2381_XI_4
2381_XI_8
2381_XI_12
2381_XI_7
2381_XI_3
2381_XI_1
2381_XI_11
2381_XI_6
2381_XI_2
2381_IX_6
2381_IX_5
2381_IX_12
2381_IX_1
2381_IX_10
2381_IX_9
2381_IX_11
2381_IX_7
2381_IX_4
2381_IX_3
2381_IX_8
2381_VIII_8
2381_VIII_9
2381_VIII_12
2381_VIII_2
2381_VIII_4
2381_VIII_10
2381_VIII_6
0.004
2386_VI_3
2386_VI_7
2386_VI_12
2386_VI_9
2386_VI_8
2386_VII_7
2386_VII_4
2386_VII_9
2386_VII_3
2386_VII_10
2386_VII_2
2386_VIII_11
2386_VIII_4
2386_VIII_8
2386_VIII_6
0.009
2533_V_6
2533_V_2
2533_V_11
2533_V_4
2533_V_3
2533_V_8
2533_V_5
2533_VI_6
2533_VI_11
2533_VII_11
2533_VII_10
2533_VII_3
2533_VII_1
2533_VII_2
2533_VII_6
2533_VII_8
2533_VII_5
2533_VII_4
2533_VII_7
2533_VII_12
2533_VII_9
2533_VI_7
2533_VI_1
2533_I_11
2533_I_8
2533_I_12
2533_I_10
2533_I_9
2533_I_5
2533_I_2
2533_I_7
2533_I_1
2533_I_6
0.008

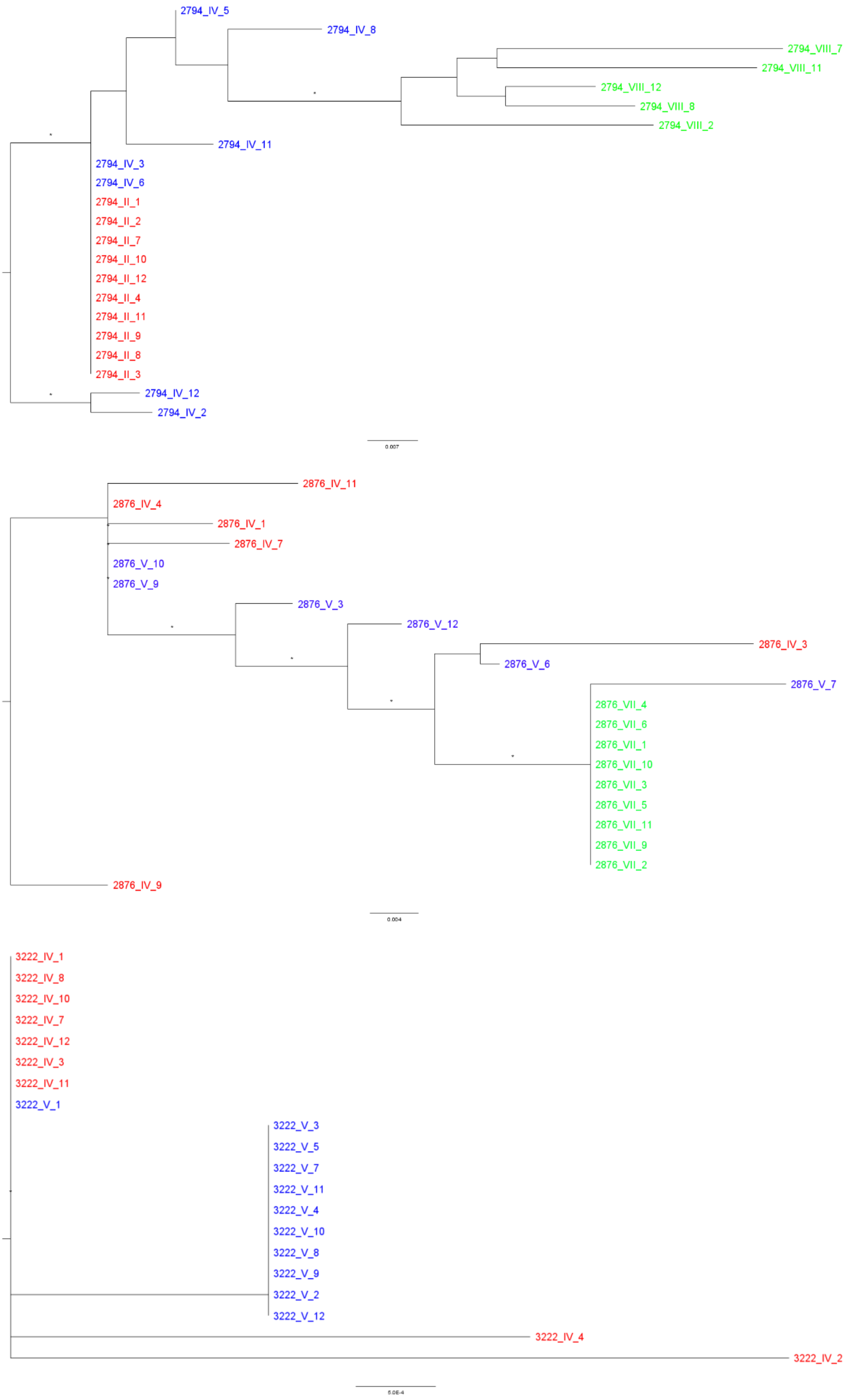
2794_IV_5
2794_IV_8
2794_VIII_7
2794_VIII_11
2794_VIII_12
2794_VIII_8
2794_VIII_2
2794_IV_11
2794_IV_3
2794_IV_6
2794_II_1
2794_II_2
2794_II_7
2794_II_10
2794_II_12
2794_II_4
2794_II_11
2794_II_9
2794_II_8
2794_II_3
2794_IV_12
2794_IV_2
0.007
2876_IV_11
2876_IV_4
2876_IV_1
2876_IV_7
2876_V_10
2876_V_9
2876_V_3
2876_V_12
2876_IV_3
2876_V_6
2876_V_7
2876_VII_4
2876_VII_6
2876_VII_1
2876_VII_10
2876_VII_3
2876_VII_5
2876_VII_11
2876_VII_9
2876_VII_2
2876_IV_9
0.004
3222_IV_1
3222_IV_8
3222_IV_10
3222_IV_7
3222_IV_12
3222_IV_3
3222_IV_11
3222_V_1
3222_V_3
3222_V_5
3222_V_7
3222_V_11
3222_V_4
3222_V_10
3222_V_8
3222_V_9
3222_V_2
3222_V_12
3222_IV_4
3222_IV_2
5.0E-4

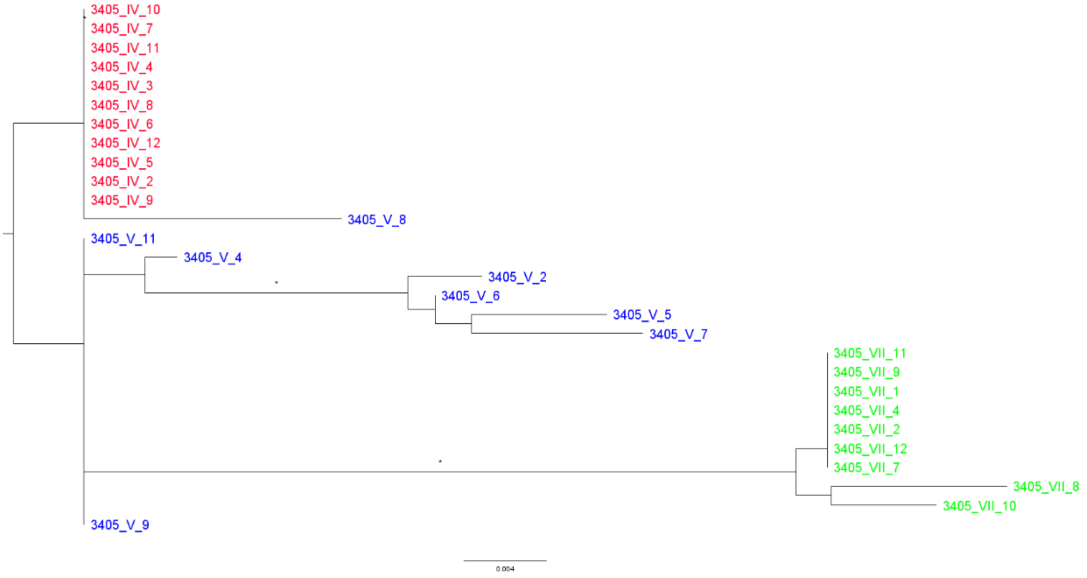
3405_IV_10
3405_IV_7
3405_IV_11
3405_IV_4
3405_IV_3
3405_IV_8
3405_IV_6
3405_IV_12
3405_IV_5
3405_IV_2
3405_IV_9
3405_V_8
3405_V_11
3405_V_4
3405_V_2
3405_V_6
3405_V_5
3405_V_7
3405_VII_11
3405_VII_9
3405_VII_1
3405_VII_4
3405_VII_2
3405_VII_12
3405_VII_7
3405_VII_8
3405_VII_10
3405_V_9
0.004

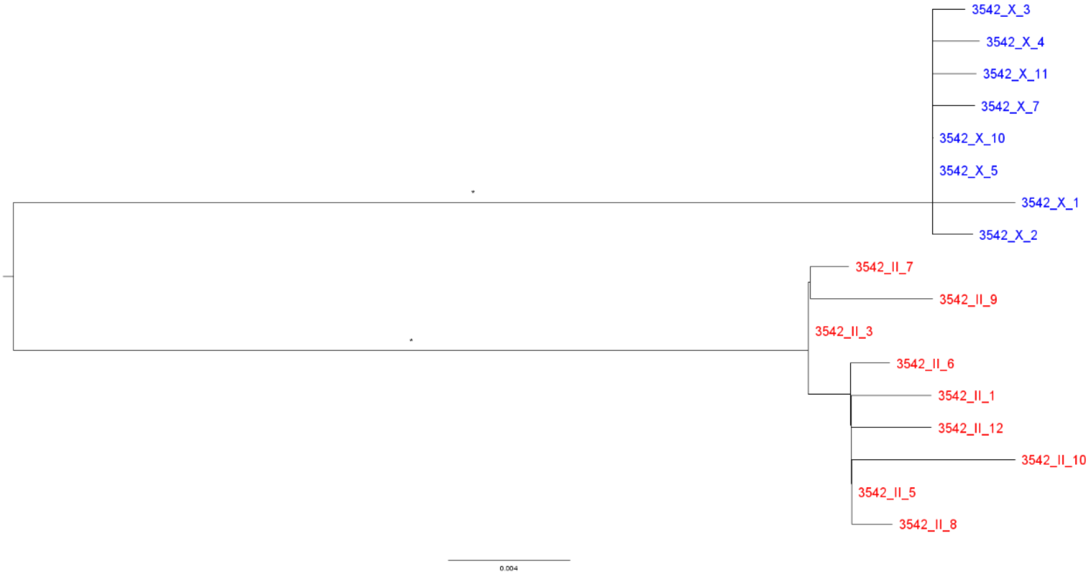
3542_X_3
3542_X_4
3542_X_11
3542_X_7
3542_X_10
3542_X_5
3542_X_1
3542_X_2
3542_II_7
3542_II_9
3542_II_3
3542_II_6
3542_II_1
3542_II_12
3542_II_10
3542_II_5
3542_II_8
0.004

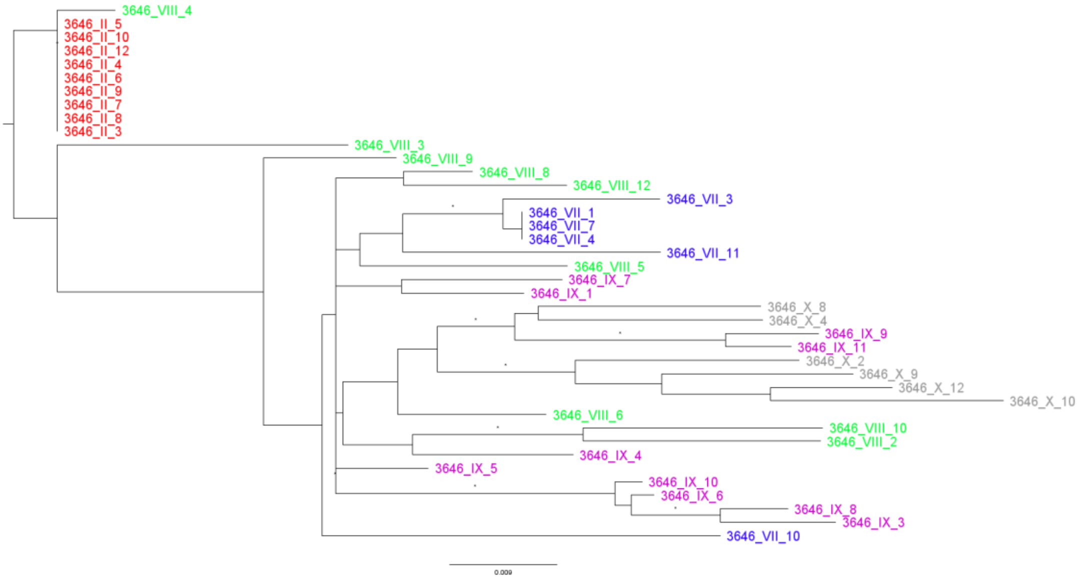
3646_VIII_4
3646_II_5
3646_II_10
3646_II_12
3646_II_4
3646_II_6
3646_II_9
3646_II_7
3646_II_8
3646_II_3
3646_VIII_3
3646_VIII_9
3646_VIII_8
3646_VIII_12
3646_VII_3
3646_VII_1
3646_VII_7
3646_VII_4
3646_VII_11
3646_VIII_5
3646_IX_7
3646_IX_1
3646_X_8
3646_X_4
3646_IX_9
3646_IX_11
3646_X_2
3646_X_9
3646_X_12
3646_X_10
3646_VIII_6
3646_VIII_10
3646_VIII_2
3646_IX_4
3646_IX_5
3646_IX_10
3646_IX_6
3646_IX_8
3646_IX_3
3646_VII_10
0.009

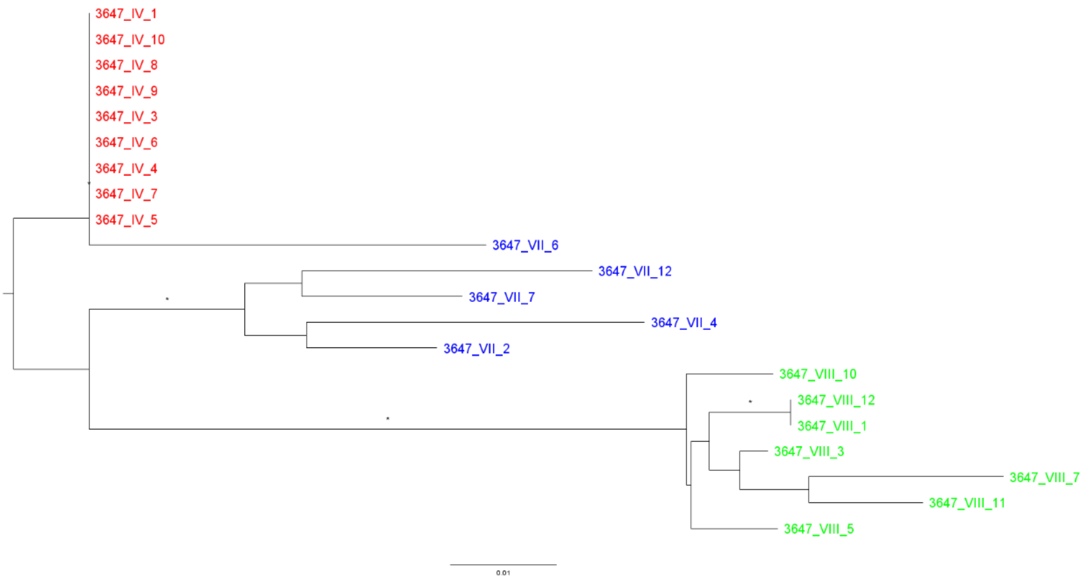
3647_IV_1
3647_IV_10
3647_IV_8
3647_IV_9
3647_IV_3
3647_IV_6
3647_IV_4
3647_IV_7
3647_IV_5
3647_VII_6
3647_VII_12
3647_VII_7
3647_VII_4
3647_VII_2
3647_VIII_10
3647_VIII_12
3647_VIII_1
3647_VIII_3
3647_VIII_7
3647_VIII_11
3647_VIII_5
0.01

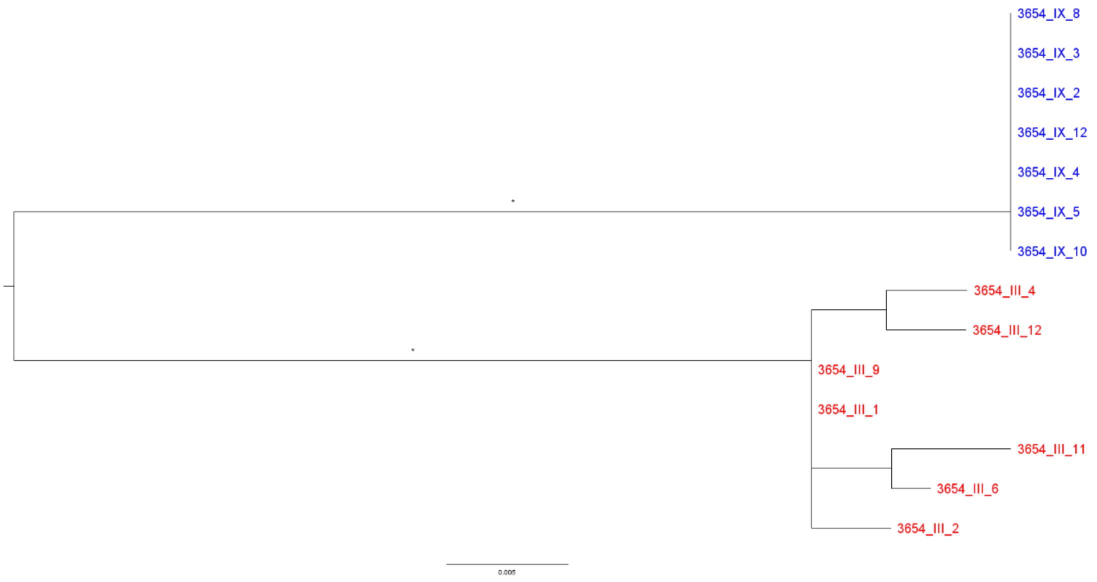
3654_IX_8
3654_IX_3
3654_IX_2
3654_IX_12
3654_IX_4
3654_IX_5
3654_IX_10
3654_III_4
3654_III_12
3654_III_9
3654_III_1
3654_III_11
3654_III_6
3654_III_2
0.005

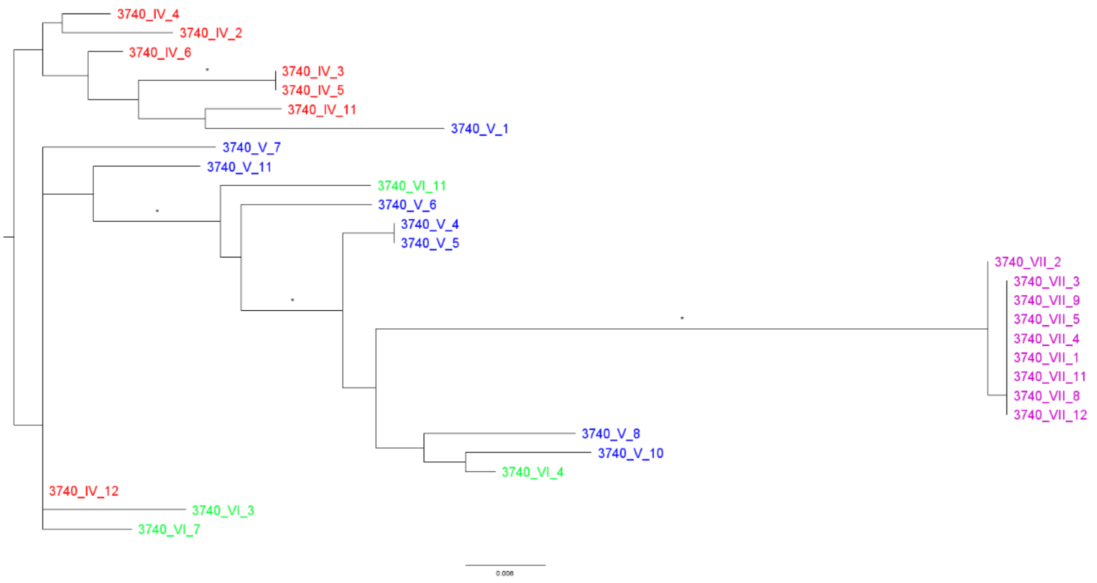
3740_IV_4
3740_IV_2
3740_IV_6
3740_IV_3
3740_IV_5
3740_IV_11
3740_V_1
3740_V_7
3740_V_11
3740_VI_11
3740_V_6
3740_V_4
3740_V_5
3740_VII_2
3740_VII_3
3740_VII_9
3740_VII_5
3740_VII_4
3740_VII_1
3740_VII_11
3740_VII_8
3740_VII_12
3740_V_8
3740_V_10
3740_VI_4
3740_IV_12
3740_VI_3
3740_VI_7
0.006

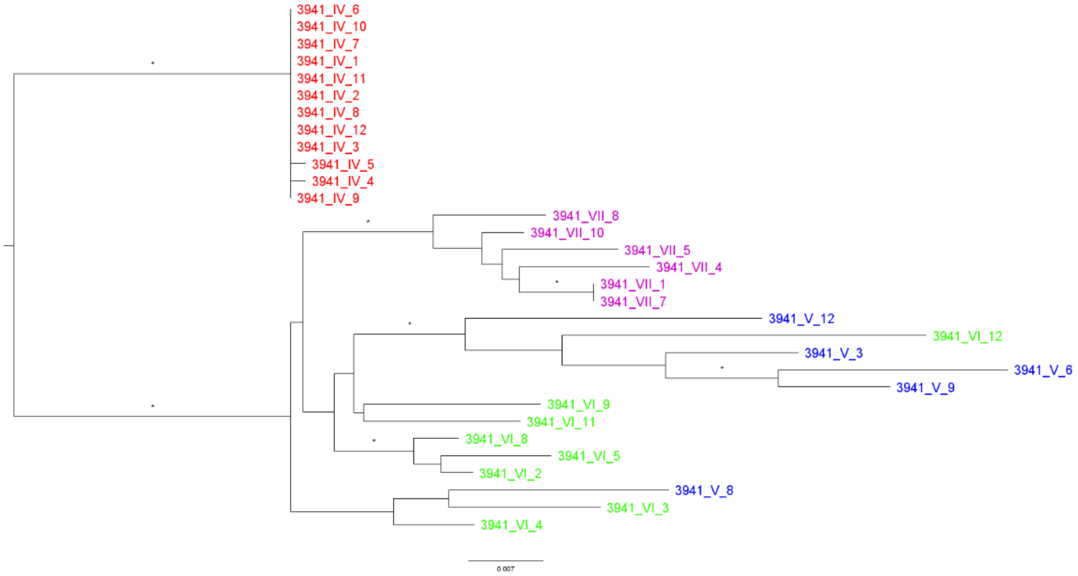

Figure S1. Maximum-likelihood (ML) phylogenetic trees reconstructed for each individual separately. The ML-based approximate likelihood ratio test (aLRT) Shimodaira-Hasegawa (SH)-like branch support was used to assess statistical support for internal branches. SH-values above 0.9 were considered statistically significant, indicated by asterisks in the trees. The analyzed samples are color coded as follows: red – first timepoint, blue – second timepoint, green – third timepoint, purple – forth timepoint, grey – fifth timepoint.

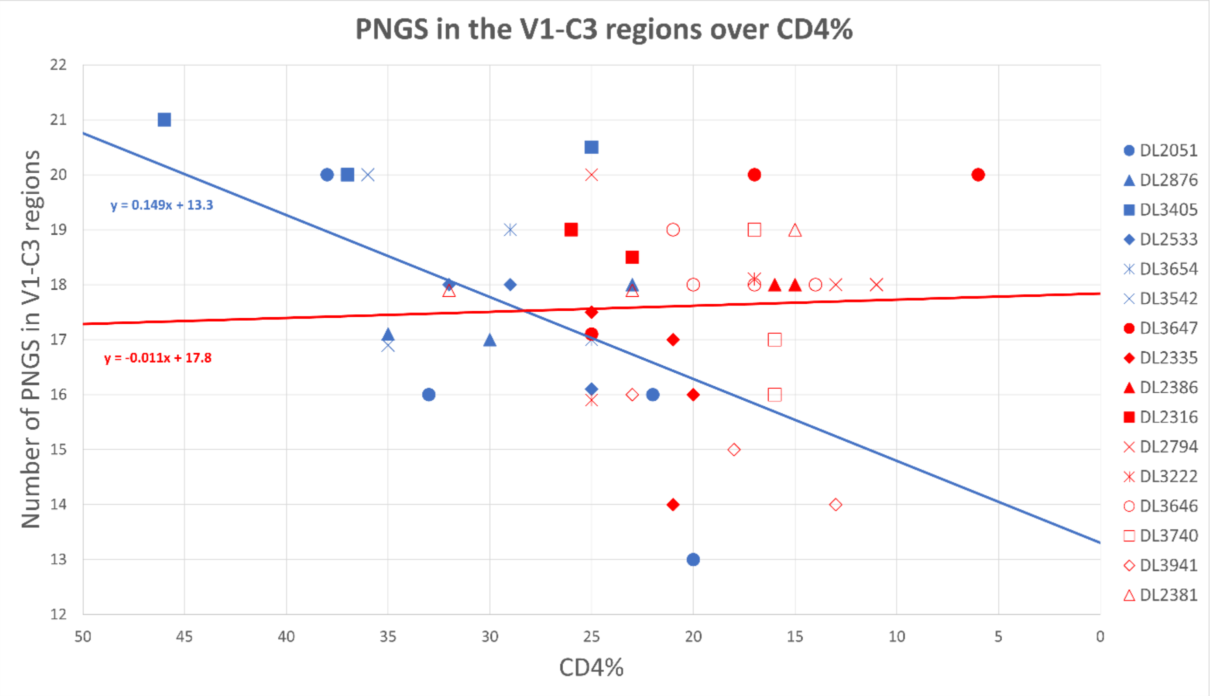

Figure S2. Linear mixed model analysis of the number of PNGS in the V1-C3 regions as a function of CD4% as a continuous fixed effect and progressor group (faster and slower progressors) as a categorical fixed effect. The interaction term between CD4% and progressor group was included to allow for a different relationship between PNGS and CD4% in faster and slower progressors. Patient was included as random effect, with the level (intercept) of PNGS (i.e. the dependent variable) allowed to vary between patients. Each data point included in the analysis are presented in the plot, with different markers for each individual. Faster progressors are colored in red and slower progressors in blue. The regression lines represent the group specific model estimates for the linear relationship between PNGS and CD4%.

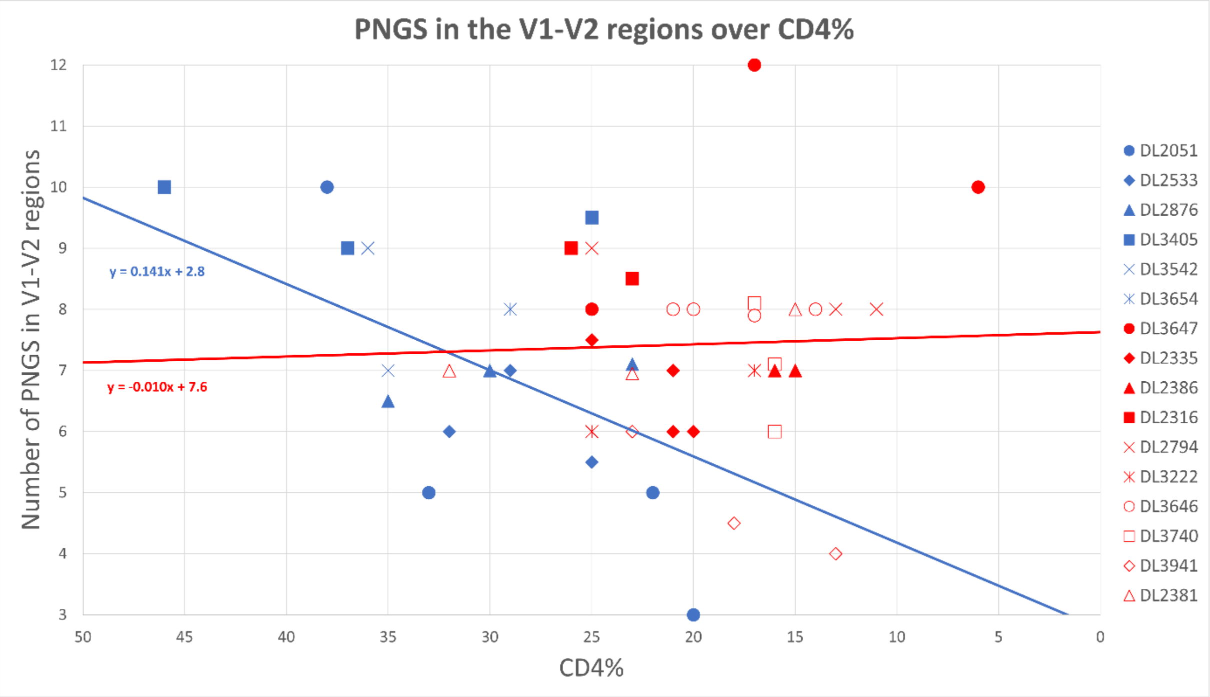

Figure S3. Linear mixed model analysis of the number of PNGS in the V1-V2 regions as a function of CD4% as a continuous fixed effect and progressor group (faster and slower progressors) as a categorical fixed effect. The interaction term between CD4% and progressor group was included to allow for a different relationship between PNGS and CD4% in faster and slower progressors. Patient was included as random effect, with the level (intercept) of PNGS (i.e. the dependent variable) allowed to vary between patients. Each data point included in the analysis are presented in the plot, with different markers for each individual. Faster progressors are colored in red and slower progressors in blue. The regression lines represent the group specific model estimates for the linear relationship between PNGS and CD4%.

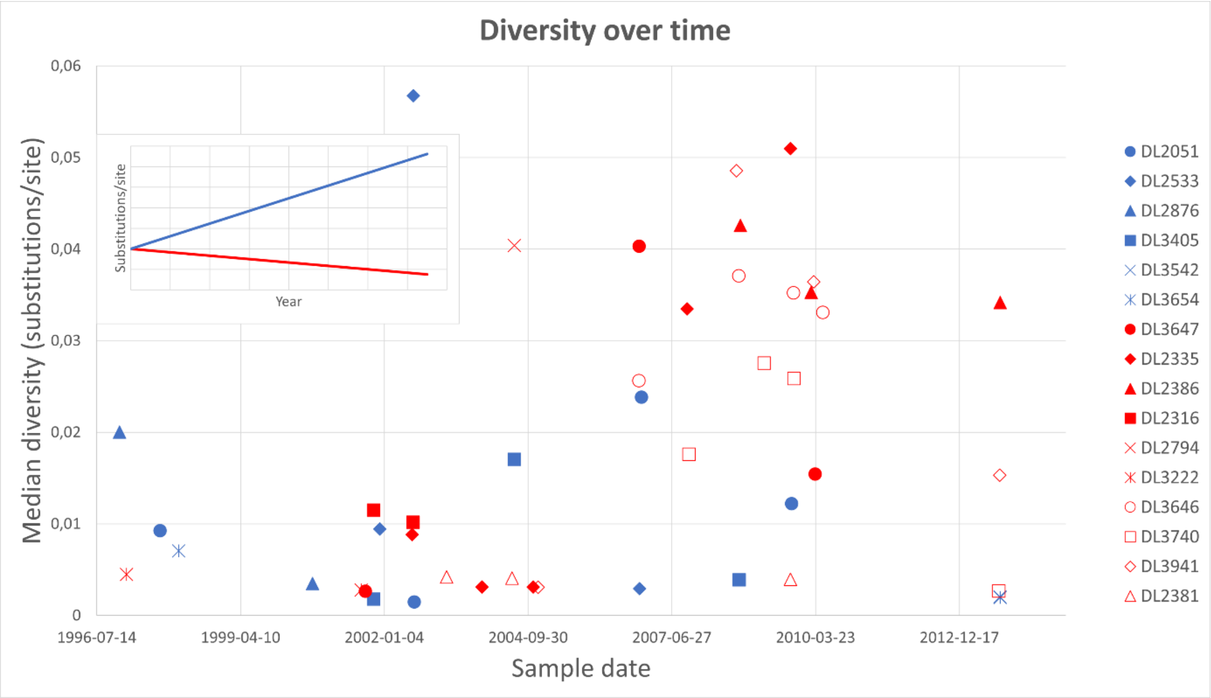

Figure S4. Linear mixed model analysis of the median diversity as a function of sample date as a continuous fixed effect and progressor group (faster and slower progressors) as a categorical fixed effect. The interaction term between sample date and progressor group was included to allow for a different relationship between diversity and CD4% in faster and slower progressors. Patient was included as random effect, with the level (intercept) of diversity (i.e. the dependent variable) allowed to vary between patients. Each data point included in the analysis are presented in the plot, with different markers for each individual. Faster progressors are colored in red and slower progressors in blue. Since the timing of sampling in relation to disease progression were not aligned between patients we did not test for differences in intercept. However, mixed model estimates of the rate of change in diversity with time (i.e. slope) did differ between progressor groups and was estimated to -4.15×10^{-4} for slower progressors and 1.54×10^{-3} for faster progressors (as illustrated in the small inset figure in the top left corner).

Table S1. Baseline characteristics of the study participants.

Individual	Sex	Age at inclusion	Observation time[a]	Date of inclusion[b]	Date of infection[c]	Date of AIDS	Time between follow-up visits[d]
DL3405	M	28	188	10-Feb-1993	26-Jun-1999		24
DL3542	M	29	241	18-Aug-1993		13-Nov-2007	22
DL2051	M	28	252	13-Feb-1990		24-Feb-2011	25
DL2876	F	41	174	20-May-1992		07-Dec-2005	19
DL3654	F	25	238	16-Nov-1993	02-Jul-1997		24
DL2533	M	25	201	19-Apr-1991		16-Jan-2008	25
DL2316	M	26	149	17-Jul-1990	08-Jul-1991	22-Jul-2002	15
DL2794	M	32	154	29-Aug-1991		26-Jun-2004	19
DL3941	M	27	226	22-Nov-1994	12-Jun-2000	23-Sep-2013	32
DL2381	M	29	278	19-Jul-1990			23
DL2335	M	33	278	17-Jul-1990		29-Sep-2009	28
DL3647	M	30	196	10-Nov-1993		03-Dec-2003	28
DL3646	M	38	238	10-Nov-1993			24
DL3222	M	37	178	28-Aug-1992	18-May-1994		30
DL3740	M	39	236	25-Jan-1994	10-Jul-2004		21
DL2386	M	30	278	19-Jul-1990	29-Jul-1995		31

[a]The median observation time (in months) from inclusion into the cohort until end-point.
[b]Date of inclusion into the cohort. Seroprevalent individuals were HIV-2 infected at the time of inclusion whereas seroincident individuals were HIV negative at inclusion and subsequently became HIV-2 infected at the estimated date of infection.
[c]Estimated dates of infection, defined as the midpoint between the last HIV-2 seronegative and the first seropositive sample.
[d]The average time (in months) between samples, determined as the time from the first to last sample, divided by the number of follow-up visits.

Table S2. Pairwise comparisons of number of PNGS per amino acid in the different regions.

Comparison	p^a	Median PNGS first group	Median length (aa) first group	Median PNGS/aa first group	Median PNGS second group	Median length (aa) second group	Median PNGS/aa second group
V1-V2 vs C2	1.000	7	94	0.08	8	99	0.08
V1-V2 vs V3	0.003	Red.	Red.	Red.	1	34	0.03
V1-V2 vs C3	0.003	Red.	Red.	Red.	2	58	0.03
C2 vs V3	0.001	Red.	Red.	Red.	Red.	Red.	Red.
C2 vs C3	0.003	Red.	Red.	Red.	Red.	Red.	Red.
V3 vs C3	1.000	Red.	Red.	Red.	Red.	Red.	Red.

[a]Significant Friedmans test was followed by Bonferroni corrected Wilcoxon singed rank test for pairwise comparisons between regions.

aa - amino acid

Red. - Redundant value presented previously in the table

Table S3. Mixed model estimates of mean diversity, PNGS, fragment length and fragment charge at a CD4% level corresponding to 35% and 14% for faster and slower progressors stratified based on CD4% decline rate and mean rate of change of these properties with change in CD4% (slope).

	**Slope**[a]			**Slope**[b]		**CD4% = 35**[c]			**CD4% = 14**[c]		
**Property**	**Slower**	**Faster**	***p***	**All individuals**	***p***	**Slower**	**Faster**	***p***	**Slower**	**Faster**	***p***
Diversity	$-6.45x10^{-4}$	$-2.36x10^{-4}$	0.472	$-4.26x10^{-4}$	0.135	0.011	0.013	0.752	0.020	0.021	0.752
V1-C3 PNGS	0.077	0.039	0.557	0.080	0.047*	19.297	17.987	0.049*	17.623	16.312	0.049*
V1-V2 PNGS	0.039	0.048	0.880	0.067	0.061	8.401	7.687	0.277	6.988	6.274	0.277
C2 PNGS	0.001	0.007	0.757	0.005	0.613	7.922	7.782	0.466	7.822	7.681	0.466
V3 PNGS[d]	-	-	-	-	-	-	-	-	-	-	-
C3 PNGS	0.027	0.001	0.159	0.012	0.204	1.874	1.691	0.414	1.630	1.447	0.414
V1-C3 length	0.218	-0.123	0.166	0.013	0.911	285.162	284.714	0.858	284.881	284.433	0.858
V1-V2 length	0.128	-0.097	0.313	0.005	0.963	93.798	93.813	0.994	93.695	93.710	0.994
C2 length[d]	-	-	-	-	-	-	-	-	-	-	-
V3 length[d]	-	-	-	-	-	-	-	-	-	-	-
C3 lenght[d]	-	-	-	-	-	-	-	-	-	-	-
V1-C3 charge	0.028	-0.110	0.063	-0.043	0.237	6.637	7.481	0.291	7.546	8.389	0.291
V1-V2 charge	-0.001	-0.082	0.110	-0.049	0.054	-3.972	-3.530	0.521	-2.939	-2.497	0.521
C2 charge	0.066	0.001	0.061	0.027	0.122	3.640	3.737	0.825	3.073	3.170	0.825
V3 charge	-0.026	0.011	0.228	-0.006	0.565	5.245	5.428	0.627	5.372	5.555	0.627
C3 charge	-0.061	-0.047	0.848	-0.049	0.162	1.074	1.483	0.521	2.111	2.520	0.521

[a]Results of mixed model analysis including the interaction between CD4% and progressor group, i.e. the model includes a separate slope for the relationship between the property and increasing CD4% for each group. The *p*-value refers to the test of the interaction, i.e. if the relationship between the property and CD4% differ between groups.
[b]Results of mixed model analysis excluding the interaction between CD4% and progressor group, i.e. the model estimates a common slope for the linear relationship between property and increasing CD4% for all individuals of both groups. This analysis was only performed if the interaction was not significant. The *p*-value refers to the test of the common slope versus zero, i.e if the property changes significantly with decreasing CD4%.
[c]Mixed model estimated means for the property at CD4% = 35 and CD4% = 14 for each progressor group. The *p*-value refers to the test of differences in mean between groups. If the interaction term for the complete model was significant, reported values are from the complete model. If the interaction was not significant, reported values are from the simplified model without the interaction. In the latter case, the net difference at both CD4% levels, and the *p*-values for the tests, will be identical.
[d]Analysis not performed due to limited variation of that property between samples.
*Denotes significant *p*-value.

Table S4. Mixed model estimates of mean rates of change per year of diversity, PNGS, fragment length and fragment charge with sampling date (slope) in faster and slower progressors stratified by CD4% decline rate.

Property	Slope[a]			Slope[b]	
	**Slower**	**Faster**	***p***	**All individuals**	***p***
Diversity	$6.94x10^{-4}$	$8.54x10^{-4}$	0.831	$7.73x10^{-4}$	0.042*
V1-C3 PNGS	$-3.07x10^{-2}$	$8.82x10^{-3}$	0.632	$-1.13x10^{-2}$	0.783
V1-V2 PNGS	$-1.57x10^{-2}$	$-1.50x10^{-2}$	0.992	$-1.54x10^{-2}$	0.684
C2 PNGS	$-2.69x10^{-4}$	$4.62x10^{-3}$	0.831	$2.12x10^{-3}$	0.852
V3 PNGS[c]	-	-	-	-	-
C3 PNGS	$-1.20x10^{-2}$	$1.66x10^{-2}$	0.198	$1.79x10^{-3}$	0.872
V1-C3 length	$6.45x10^{-2}$	$2.03x10^{-1}$	0.561	$1.33x10^{-1}$	0.265
V1-V2 length	$6.01x10^{-2}$	$1.82x10^{-1}$	0.602	$1.20x10^{-1}$	0.303
C2 length[c]	-	-	-	-	-
V3 length[c]	-	-	-	-	-
C3 length[c]	-	-	-	-	-
V1-C3 charge	$2.51x10^{-3}$	$-2.63x10^{-2}$	0.764	$-8.91x10^{-3}$	0.852
V1-V2 charge	$2.37x10^{-2}$	$7.57x10^{-2}$	0.435	$4.94x10^{-2}$	0.139
C2 charge	$-3.07x10^{-3}$	$-1.50x10^{-2}$	0.761	$-8.77x10^{-3}$	0.651
V3 charge	$-1.38x10^{-2}$	$-1.71x10^{-2}$	0.900	$-1.54x10^{-2}$	0.237
C3 charge	$3.50x10^{-2}$	$-2.15x10^{-3}$	0.546	$1.68x10^{-2}$	0.581

[a]Results of mixed model analysis including the interaction between time and progressor group, i.e. the model includes a separate slope for the relationship between the property and time for each group. The *p*-value refers to the test of the interaction, i.e. if the relationship between the property and time differ between groups.
[b]Results of mixed model analysis excluding the interaction between time and progressor group, i.e. the model estimates a common slope for the linear relationship between property and time for all individuals of both groups. This analysis was only performed if the interaction was not significant. The *p*-value refers to the test of the common slope versus zero, i.e if the property changes significantly with time.
[c]Analysis not performed due to limited variation of that property between samples.
*Denotes significant *p*-value.
